# Treatment of major depressive disorder with bilateral theta burst stimulation: study protocol for a randomized, double-blind, placebo-controlled multicenter trial (TBS-D)

**DOI:** 10.1007/s00406-021-01280-w

**Published:** 2021-06-19

**Authors:** Christian Plewnia, Bettina Brendel, Tobias Schwippel, Vanessa Nieratschker, Thomas Ethofer, Thomas Kammer, Frank Padberg, Peter Martus, Andreas J. Fallgatter

**Affiliations:** 1 Department of Psychiatry and Psychotherapy, Brain Stimulation Center, Tübingen Center for Mental Health (TüCMH), Neurophysiology and Interventional Neuropsychiatry, University of Tübingen, Calwerstrasse 14, 72076 Tübingen, Germany; 2 Institute of Clinical Epidemiology and Applied Biostatistics (IKEaB), University of Tübingen, Tübingen, Germany; 3 Department of Biomedical Magnetic Resonance, University of Tübingen, Tübingen, Germany; 4 Department of Psychiatry and Psychotherapy, University of Ulm, Ulm, Germany; 5 Department of Psychiatry and Psychotherapy, LMU Hospital, Munich, Germany

**Keywords:** Major depression, Transcranial magnetic stimulation, Theta burst stimulation, Multicenter, Randomized controlled trial, Brain stimulation

## Abstract

Repetitive transcranial magnetic stimulation (rTMS) of the dorsolateral prefrontal cortex (dlPFC) is currently evolving as an effective and safe therapeutic tool in the treatment of major depressive disorder (MDD). However, already established rTMS treatment paradigms are rather time-consuming. With theta burst stimulation (TBS), a patterned form of rTMS, treatment time can be substantially reduced. Pilot studies and a randomized controlled trial (RCT) demonstrate non-inferiority of TBS to 10 Hz rTMS and support a wider use in MDD. Still, data from placebo-controlled multicenter RCTs are lacking. In this placebo-controlled multicenter study, 236 patients with MDD will be randomized to either intermittent TBS (iTBS) to the left and continuous TBS (cTBS) to the right dlPFC or bilateral sham stimulation (1:1 ratio). The treatment will be performed with 80% resting motor threshold intensity over six consecutive weeks (30 sessions). The primary outcome is the treatment response rate (Montgomery-Asberg Depression Rating Scale reduction ≥ 50%). The aim of the study is to confirm the superiority of active bilateral TBS compared to placebo treatment. In two satellite studies, we intend to identify possible MRI-based and (epi-)genetic predictors of responsiveness to TBS therapy. Positive results will support the clinical use of bilateral TBS as an advantageous, efficient, and well-tolerated treatment and pave the way for further individualization of MDD therapy.

Trial registration: ClinicalTrials.gov (NCT04392947).

## Background

Major depressive disorder (MDD) is one of the leading causes of disease burden, severely impairs quality of life, autonomy, social integration, and life expectancy. Although psychotherapy and medication are effective treatments, a large proportion of patients do not tolerate or sufficiently respond to the initial treatment [1]. Moreover, approximately 50% will experience a recurrent or chronic course of illness for which long-term treatment is recommended [2]. Therefore, the implementation of additional effective and tolerable interventions is highly desirable to expand and individualize treatment options.

In the last two decades, modern noninvasive brain stimulation (NIBS) techniques, particularly repetitive transcranial magnetic stimulation (rTMS), have emerged as effective new means of MDD treatment [3, 4]. Treatment effects are mediated by several mechanisms of action. Among them, left-sided high frequency-rTMS to the left dlPFC increases metabolic activity in connected areas whereas right-sided low frequency-rTMS decreased it [5–7]. In addition, it is suggested that rTMS modulates 5HT_2A_ up- and downregulation in the prefrontal areas and the hippocampus [8]. There are also indications that other neurotransmitter systems (GABA, glutamate) play a role in the effectiveness of rTMS. These different mechanisms lead to changes in functional connectivity in different brain networks [9], which are associated with clinical improvement [10]. Taken together, rTMS treatment strategies of MDD aim at a targeted modulation of dysfunctional cortical activity in prefrontal cortical regions, i.e. the dorsolateral prefrontal cortex (dlPFC) resulting in altered regulation of the respective networks involving subcortical hub regions (e.g. amygdala, subgenual cingulate, and insula) [3, 11–13]. On the behavioral level, the brain network dysfunction is reflected by a preferential perception and processing of negative information subserving the generation and maintenance of depressive symptomatology [14]. Therefore, the targeted modulation of this network by rTMS most likely represents a central neuronal mechanism underlying the beneficial effects of rTMS [15]. Recent meta-analyses agree on medium effect size in respect to response and remission [3, 16]. However, the number of multicenter trials is still rather small and positive full-scaled European trials are missing completely. Moreover, current European treatment guidelines mention rTMS as a treatment option in depression but with a weak grade of recommendation [17]. Hence, this therapy is not available for most patients in Europe and is still restricted to specialized centers or private providers not the least because health insurances do not regularly cover the costs. Therefore, additional evidence for the efficacy of rTMS is required.

However, it has to be considered that lately, theta burst stimulation (TBS) has evolved as an equally effective [18] and considerably less burdening form of rTMS treatment [19]. With TBS, a patterned form of rTMS requiring significantly briefer stimulation sessions and typically lower stimulation intensities, the ‘chair time’ and stimulation-related discomfort is substantially reduced compared to conventional high-frequency rTMS [20, 21]. Moreover, the shorter treatment duration allows for increases in dosage per session, spaced stimulation protocols with multiple stimulations sessions per day, and multifocal, e.g. bilateral stimulation. The pattern-specific modulatory effects of TBS were initially demonstrated in the motor cortex [22] and subsequently investigated for prefrontal cortex regions [23]. In motor regions, continuous TBS (cTBS) predominantly reduced and intermittent TBS (iTBS) increased the excitability of the targeted cortical neurons [24].

However, only a limited number of case series [25–27] and sham-controlled pilot trials with mixed results [28–31] exist investigating TBS of prefrontal cortex regions in terms of its efficacy in MDD. Based on that data, meta-analyses [3, 19, 32, 33] indicate that active TBS (unilateral and bilateral, 2–6 weeks of treatment) is associated with significantly higher response rates when compared to sham TBS. In addition, a large (*n* = 414) multicenter trial demonstrated the non-inferiority of iTBS compared to standard 10 Hz rTMS applied to the left dlPFC [18]. Together, these pilot-studies, meta-analyses and the non-inferiority study support the efficacy of TBS in the treatment of MDD [3, 34]. However, large-scale randomized placebo-controlled clinical trials, especially of bilateral TBS, are still needed [35]. Particularly against the backdrop of the hesitant use of rTMS as a routine depression treatment in Europe, the positive results of the large non-inferiority study [18] are still not sufficient to establish TBS for broader clinical application.

Therefore, the main objective of this clinical trial is to test the efficacy of once-daily, bilateral TBS (cTBS to the right dlPFC and iTBS to the left dlPFC) in a 6 weeks treatment of MDD as add-on to stable ongoing standard therapy. We hypothesize that the number of responders will be significantly higher within the active compared to the placebo TBS group. In addition, we aim to identify biomarkers that allow predicting which patients are most likely to respond to TBS. This aim will be addressed in two associated satellite research projects:

First, by the acquisition of magnetic resonance imaging (MRI) data, we will investigate whether structural MRI as assessed by volumetric T1-weighted images, fiber architecture examined by diffusion MRI or functional connectivity obtained in resting-state functional MRI can be used to predict treatment responses. Based on previous results [36], we hypothesize the dorsolateral prefrontal cortex (dlPFC) as well as the subgenual and rostral anterior cingulate cortex (ACC) as the potential target areas for treatment prediction. We aim to investigate which of the above-mentioned MRI modalities is best suited for treatment prediction and to clarify if their combination by means of multivariate pattern analysis can further improve prediction accuracy.

Second, based on knowledge of genetic predictors of stimulation effects [37–39], it is reasonable to assume that response to bilateral TBS can partially be predicted by multiomics factors, including genetics, epigenetics, and gene expression. However, the systematic investigation of the role of genetic factors as well as the epigenetic regulation of gene expression on TBS response is entirely missing to date. The identification of objective biomarkers with predictive value for treatment success would be highly valuable for clinicians and patients to help tailor personalized treatment options which foster treatment response and long-term remission. For patients that are most likely not benefiting from TBS, other treatment options could be considered early. Having reliable diagnostic biomarkers will hone the efficiency of stimulation treatment for depression and help to minimize treatment costs by choosing effective treatment options for the individual patient.

## Design and methods

This prospective study is designed as a multicenter, randomized, double-blind, placebo-controlled clinical trial and aims to demonstrate the superiority of bilateral theta burst stimulation compared to a placebo condition (i.e. sham TBS) over a 6 weeks treatment period (30 sessions, five per week, daily from Monday to Friday) in patients with MDD.

Altogether 236 patients will be randomly assigned (1:1 ratio) to the two parallel treatment arms. Patients will receive either active combined iTBS/cTBS, or sham iTBS/sham cTBS, respectively. In each session, cTBS will be applied to the right and iTBS to the left dlPFC successively. Active and sham stimulation will be applied as add-on treatment to stable ongoing standard care (psychopharmacological and/or psychotherapeutic therapy). Study assessment with the observer- and self-ratings will be conducted for screening and baseline, after the 2nd, 4th, and 6th week of treatment, and during the follow-up period at two time points (1 and 3 months after end of treatment period).

Two associated satellite projects, entitled ‘MRI-based prediction of TBS-treatment efficacy in MDD’, and ‘Multiomics-analysis based prediction of TBS-treatment efficacy in MDD’ focus on the identification of potential treatment predictors.

This detailed description of the study protocol follows the recommendations of the SPIRIT statement [40].

### Ethics, consent, and registration

Our study will be conducted in accordance with the principles of the Declaration of Helsinki and comply with the guidelines of Good Clinical Practice of the International Conference on Harmonization of Technical Requirements for Registration of Pharmaceuticals for Human Use (ICH). The protocol, patient information, and consent form of this clinical study and its satellite projects have been approved by the Ethics Committee of the Medical Faculty of the University of Tübingen (protocol version 1.0, approved on 31 March 2020 (097/2020BO1) with 1st (22 July 2020) and 2nd (24 February 2021) amendment) and the responsible Ethics Committees of the participating centers. Any significant modifications to the protocol (i.e. concerning study aims, stimulation procedure, inclusion and exclusion criteria, and sample size) will require an amendment that has to be approved by the Ethics Committees of all participating trial sites.

The trial has been registered on http://clinicaltrial.gov (NCT04392947). Trial registration entries will be updated regularly.

### Study population

The clinical trial will include in- and outpatients aged from 18 to 70 years with a primary diagnosis of major depression. Experienced study investigators will perform a clinical interview based on the Diagnostic and Statistical Manual of Mental Disorders 5th Edition (DSM-5). We exclude patients with a duration of illness > 2 years to reduce sample heterogeneity. Table 1 contains all inclusion and exclusion criteria for the present study.

**Table 1:** Inclusion and exclusion criteria of TBS-D

Inclusion criteria	
	Age between 18 and 70 years
	Moderate or severe current episode of MDD according to DSM-5 diagnostic criteria
	Duration of current episode of at least 6 weeks but not more than 2 years
	Hamilton Depression Rating Scale (HDRS-17) score of at least 18 (at screening)
	Mild to moderate pharmacological antidepressant treatment resistance according to the short form of Antidepressant Treatment History Form (ATHF-SF); defined as having failed at least one but no more than three adequate antidepressants treatments in the present episode
	Antidepressant medication must be stable at least 4 weeks before the start of treatment intervention (antidepressant medication-free patients can also be included, however patients have to fulfil the criteria of treatment resistance in the current episode)
	Ability to understand the verbal/written study information
	Ability to give consent
	Ability to answer the questions associated with (psychiatric) examination/to fill in the patient self-ratings
Exclusion criteria	
	Acute suicidality (MADRS item 10 score > 4)
	Other psychiatric disorders (except for anxiety disorder)
	Psychotic symptoms
	Intake of antiepileptic drugs or benzodiazepines (corresponding to > 1 mg lorazepam/day)
	Substance dependence or abuse in the past 3 months (with the exception of tobacco)
	Previous rTMS treatment
	Lifetime history of non-response to adequate electroconvulsive therapy (minimum of eight treatments)
	Deep brain stimulation
	History of seizures/brain surgery
	Significant and clinically relevant brain malformation or neoplasm
	Head injury/stroke/dementia or other neurodegenerative disorders
	Cardiac pacemakers, intracranial implant, or ferromagnetic parts in the cranium
	Pregnancy

### Study centers and recruitment

Besides the Department of Psychiatry and Psychotherapy of the University of Tübingen (coordinating center) six additional psychiatric departments in Germany will participate in the study. These centers must have the appropriate technical equipment (see below ‘Technical Devices’) and experienced staff (at least 6 months) regarding TMS treatment of depressive patients. Furthermore, the study protocol will be approved by the respective institutional ethics committees before site initiation.

All centers will recruit patients in their in- and outpatient clinics. In addition, all study centers can address potential candidates by contacting (1) local patient advocacy/support groups, (2) local psychiatrists/general practitioners, as well as (3) information via clinic website, social media, newsletters, local newspapers, or advertisement in public transportation.

During the initial phase of the recruitment process, i.e. the pre-screening period, a study investigator will give comprehensive verbal and written information about trial-related objective, procedures, and possible risks to each patient and if necessary to the legal representatives, too. All patients will have the opportunity to ask questions and will have enough time to consider whether they want to participate in the study. Before any study-specific measures can be administered, a signed consent form must be present.

Continuous site monitoring will ensure the early identification of recruitment problems. In cases of relevant delays, site-specific measures will be taken, e.g. additional dedicated staff, alternative organizational structures, intensified social media activity, and further involvement of local patient groups. However, in case of persistent and substantial recruitment delays, the study center will be closed and a suitable new center will be identified and initiated.

### Patient involvement

To ensure patient participation, the coordinating center cooperates with the nationwide patient advocacy group Deutsche DepressionsLiga (DDL, https://www.depressionsliga.de) and the Stiftung Deutsche Depressionshilfe (https://www.deutsche-depressionshilfe.de), which is a member of the European Alliance against Depression. Both organizations will support recruitment and help to disseminate information about the study and its results to the public. The DDL is especially dedicated to protecting the interests of patients within this study, acting as a forum for concerns and expectations related to TMS treatment.

### Study timeline

**Fig. 1:**
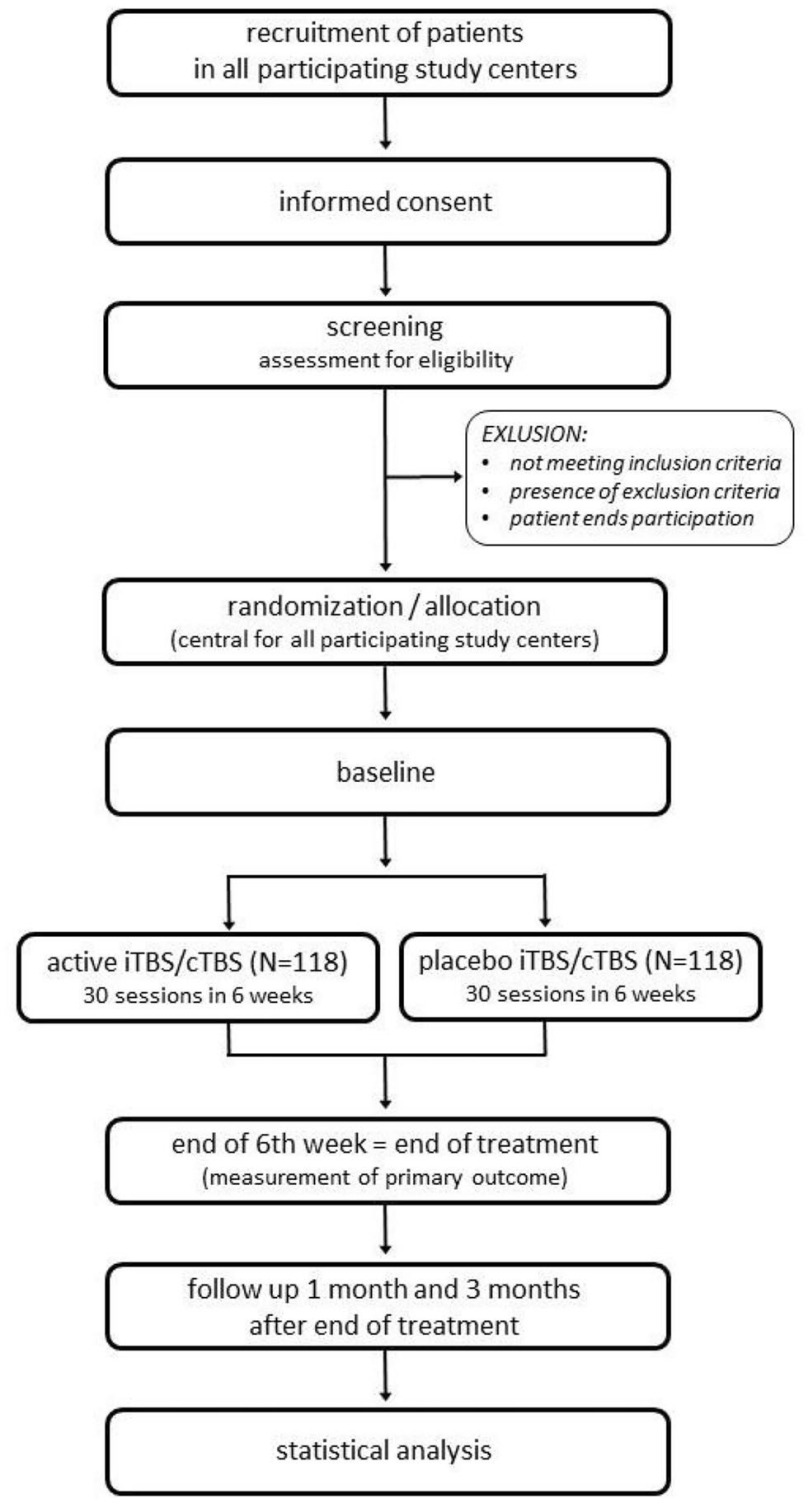
Trial flow

Figure 1 gives a overview of the study timeline. Once the signed consent form is available, the pre-treatment phase can start with the screening process (for details see “Assessment” or Table 2). As soon as it becomes evident that a patient does not meet the criteria for study participation (Table 1), all study-specific procedures will be stopped and participation in the study will end. Patients who meet the inclusion and exclusion criteria will be randomized either to the verum or placebo group. In a next step, patients will participate in the baseline session (Table 2), including MRI measurements and blood samples for patients who participate in the respective satellite-studies.

**Table 2:** Study flow chart

	Study period												
	Pre-Treatment		Treatment									Follow-up	
Week	0		1	2		3	4		5	6		10	18
Visit	Screening	Baseline	V1-V5	V6-V10	V10	V11-V15	V16-V20	V20	V21-V25	V26-V30	V30	V31	V32
Informed consent	X												
Sociodemography	X												
Pregnancy test^1^	X												
Physical/neurological examination	X												
Concomitant treatment^2^	X	X			X			X			X	X	X
SCID-5-PD	X												
ATHF-SF	X												
MADRS (item 10 only)	X												
HDRS-17	X				X			X			X	X	X
Randomization	X												
MADRS (complete)		X			X			X			X	X	X
BDI-II		X			X			X			X	X	X
CGI		X			X			X			X	X	X
WHO-5		X									X	X	X
WPAI		X									X		X
THINC-it^®^ (test of cognition)		X									X		X
CTQ		X											
EHI		X											
MRI^3^		X											
Genetics^3^		X									X		X
Determination of resting motor threshold^4^		X											
Active iTBS/cTBS sham iTBS/cTBS													
Safety check													

^1^Pregnancy test is only necessary for women in child-bearing age; if a blood sample is taken as part of the clinical routine, it is advisable to check a possible pregnancy with a blood test. Otherwise, a urine test can be used to avoid unnecessary risks

^2^Concomitant medication and/or any type of psychotherapy will be documented for each patient for his/her entire participation period

^3^MRI and Genetics are not part of the standard protocol but belong to two satellite projects

^4^Alternatively, the threshold can also be determined directly before the first stimulation, i.e. in visit 1

Study treatment will start on Monday within the week after completion of the baseline session (deviations in case of holidays are possible). During the treatment phase, each patient will receive a total of 30 TBS sessions over a period of 6 consecutive weeks (one session daily from Monday to Friday).

Ratings will take place at the end of the 2nd, 4th, and 6th weeks after the respective stimulation session.

Within the follow-up period two visits are scheduled, 1 month and 3 months after the end of treatment. Patient participation in the study will end with last follow-up.

### Assessment

#### Clinical measures

The tests and questionnaires performed during screening, baseline, visits 10, 20 and 30 and the two follow-up visits are summarized in Table 2. As such, the screening consists of (1) registration of socio-demography (age, sex, years of education), (2) documentation of concomitant treatment (medication and any type of psychotherapy), (3) physical-neurological examination, (4) psychiatric examination including differential diagnosis of MDD according to DSM-5 criteria, the Structured Clinical Interview for DSM-5 Personality Disorders (SCID-5-PD), the Antidepressant Treatment History: Short Form (ATHF-SF), the Hamilton Rating Scale for Depression—17 items (HDRS-17), and item 10 (suicidal thoughts) of Montgomery-Asberg Depression Rating Scale (MADRS) and (5) a pregnancy test if indicated (female patients in child-bearing age).

The baseline will comprise further psychopathological examination, i.e. MADRS (complete version), Clinical Global Impression Scale (CGI), Beck Depression Inventory (BDI-II), Childhood Trauma Questionnaire (CTQ), World Health Organization Five Well-Being Index (WHO-5), and Work Productivity and Activity Impairment Questionnaire (WPAI). In addition, the baseline session also includes the Edinburgh Handedness Inventory (EHI) and the THINC-Integrated Tool (THINC-it^®^), a computerized test battery for measuring cognitive functions in patients with major depression [41].

During the treatment period, visit 10 (end of 2nd week) and visit 20 (end of 4th week) comprise HRSD-17, MADRS, CGI, and BDI-II. Visit 30 (end of treatment) will additionally include WHO-5, WPAI and the repetition of the THINC-it^®^ cognitive test battery. The first follow-up is identical to visits 10 and 20. The second follow-up is identical to visit 30.

Regarding the primary endpoint (MADRS), a comprehensive rater training is delivered comprising training and a validation phase. For the training phase, three videos are available to explain the (expected) ratings of the different items. A fourth video is used to assess, i.e. whether the raters' assessments match the sample solution. Any deviations will be discussed with the principal investigator. If there are deviations of more than ± 1 point, the training must be repeated. Furthermore, instructions for raters will be improved based on these discussions and communicated to all study centers.

#### MRI acquisition

Study participants will undergo MRI prior to the first TBS intervention including acquisition of a magnetization prepared rapid acquisition gradient echo (MPRAGE) sequence to obtain high resolution T1-weighted images (TR = 2.3 s, TE = 4.16 ms, TI = 0.9 s, flip angle 9°, voxel size = 1 × 1 × 1 mm^3^), a sampling perfection with application optimized contrasts using different flip angle Evolutions (SPACE) sequence to obtain high resolution T2-weighted images (TR = 5 s, TE = 383 ms, TI = 1.8 s, voxel size = 1 × 1 × 1 mm^3^), diffusion MRI (TR = 6 s, TE = 55 ms, bandwidth: 1930 Hz/voxel, flip angle = 90°, 70 axial slices, voxel size of 2 × 2 × 2 mm^3^) along 64 independent directions using a b-value of 1500 s/mm^2^, a gradient echo fieldmap for image distortion correction (TR = 0.4 s, TE(1) = 5.19 ms, TE(2) = 7.65 ms, flip angle = 60°, 36 slices with 3 mm slice thickness + 1 mm gap, voxel size = 3 × 3 × 4 mm^3^), and functional MRI (400 images, 69 transversal slices acquired interleaved, TR = 1.5 s, TE = 30 ms, flip angle = 70°, voxel size = 2 × 2 × 2 mm^3^, multiband acceleration factor 3) obtained during resting-state with eyes-open and presentation of the movie paradigm Inscapes [42]. Inscapes is freely available via www.headspacestudios.org/inscapes and features abstract shapes without a narrative or scene-cuts and was designed to decrease head-motion and increase wakefulness while minimizing cognitive load during the acquisition of resting-state functional MRI data.

#### Multi-omics analysis

Participation in the multiomics satellite study involves the collection of a blood sample (30 ml EDTA, 2.5 ml PAXgene™) at three-time points (baseline, end of 6th week, and 2nd follow-up) to allow for genetic, epigenetic, and gene expression analyses. To investigate epigenetic markers potentially predicting therapy response, epigenome-wide analyses will be performed using the Illumina EPIC MethylationEPIC BeadChip array [43]. DNA methylation of top hits will be validated by pyrosequencing. For investigating gene expression markers whole-genome RNA-sequencing will be performed on whole blood samples. Expression levels of differentially expressed key hub and regulator genes will be validated by quantitative RT-PCR (= reverse transcription-polymerase chain reaction) using globin reduced total RNA derived from whole blood. To study the potential influences of genetic variation on epigenetic regulation and phenotypic outcome, genetic analysis of the candidate genes will be conducted.

### Randomization

The Institute of Clinical Epidemiology and Applied Biostatistics of the University of Tübingen (IKEaB) is responsible for randomization. By using the randomization tool of the software nQuery (release 8), patients will be allocated either to the active or placebo group in 1:1 ratio with varying block lengths. Additionally, the order of cTBS and iTBS in the first session (cTBS–iTBS or iTBS–cTBS, respectively) will be randomized and subsequently alternated in all following sessions. Randomization is stratified for the study center.

Technically, the IKEaB will receive a list with patient keys (6-digit numerical codes) provided by the manufacturer of the magnetic stimulator (MagVenture^©^, Farum, Denmark). These patient keys must be entered into the magnetic stimulator to start the stimulation session and determine whether the machine delivers verum or sham stimulation (for details see “Intervention”). Each patient key will be used only once during the entire trial and each randomized patient will keep his/her patient key until the end of treatment. The patient keys are matched to the randomization list obtained from the software nQuery by using the statistical software SPSS. This will be done by an independent statistician not involved in the study.

The study centers will receive only the patient keys together with the information about the order of iTBS and cTBS on demand by using the computerized randomization tool of our database secuTrial^®^. The patient key itself cannot be used to identify the treatment arm, thus ensuring blinding of study physicians, TMS-operators, patients, and data analysing statistician.

### Intervention

#### Determination of stimulation intensity

At the end of baseline/before the first stimulation session, the study physician will determine the individual stimulation intensity based on the resting motor threshold (RMT), which will be measured separately for the right and left primary motor cortex. It is defined as the lowest stimulation intensity that induces a motor response in at least five of ten TMS stimuli. According to the standard clinical practice, it will be assessed by visual observation of contralateral thumb twitches [4]. Individual stimulation intensity will be set to 80% RMT for left iTBS and right cTBS during the 6-week treatment period. Pulse form will be biphasic, with an anterior–posterior to posterior–anterior current direction. The handle of the coil will be oriented backwards rotated about 45° from sagittal orientation with the axis pointing towards the nasion.

#### Determination of targeted brain areas

The target areas for stimulation are the left and right dlPFC. Since neuronavigation is not practicable in such a large sample size, following the EEG 10–20 system is accepted as the most feasible approach to locate the specific brain areas [18, 44, 45]. We will use the beam F3 system to determine the individual stimulation targets F3 and F4 corresponding to the left dlPFC the right dlPFC, respectively. The orientation of the coil handle will be similar to the stimulation of the respective motor hot spot.

#### Stimulation: cTBS and iTBS

**Fig. 2:**
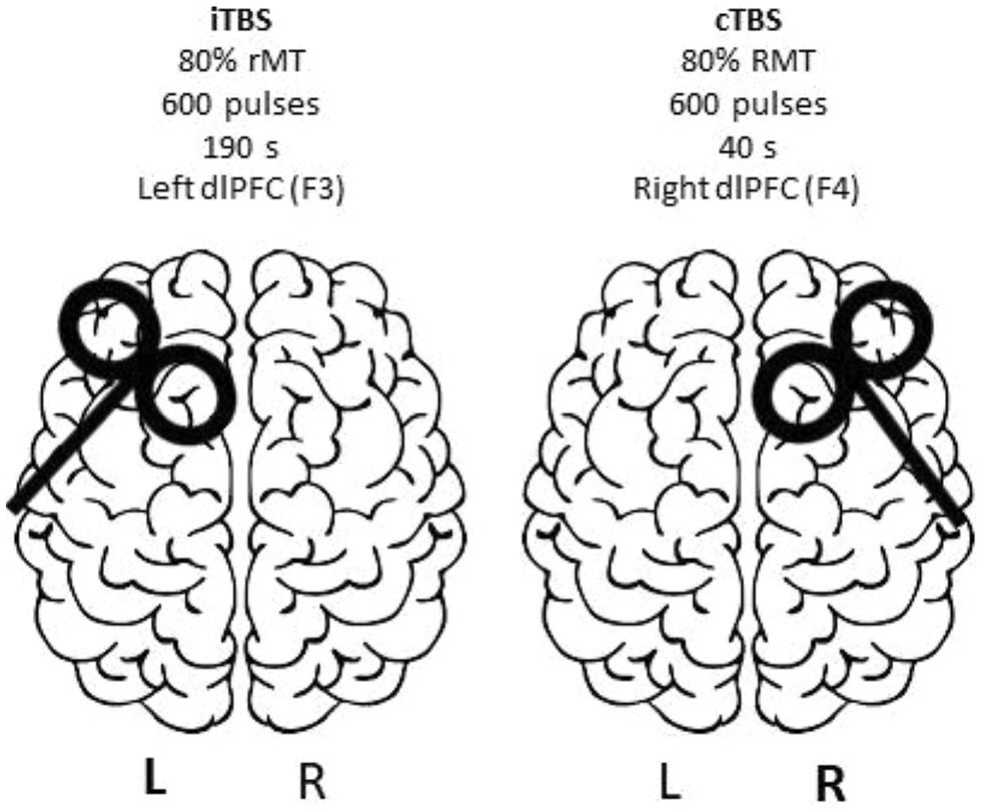
Bilateral Theta burst stimulation during TBS-D. Simplified sagittal brain drawing, TMS figure-of-eight coil over the left and right dorsolateral cortex (F3/F4, EEG 10–20 system). The coil is directed to the nasion. Modified picture taken from pixabay.com

Based on the standard TBS protocol [22], each stimulation session will comprise two trains of 600 stimuli (Fig. 2) each applied in bursts of three pulses at 50 Hz given every 200 ms. On the left-side, activity enhancing iTBS (2 s on/8 s off) will be applied 20 times (600 stimuli) for a total duration of 3 min 12 s. In the same session, cTBS targeting the right dlPFC will be applied continuously for 40 s (600 stimuli). For the first session, the order of cTBS/iTBS will be determined by randomization and will alternate in each following session to preclude order effects.

#### Technical devices

Both, the determination of the RMT as well as the active and sham TBS will be carried out with the same magnetic stimulator (MagPro X100 or R30; MagVenture^©^, Farum, Denmark). The determination of the RMT will be performed with a 150° angled figure-of-eight coil (Cool B70) and the verum or sham stimulation with the corresponding active-placebo coil (Cool B70 A/P). To activate the treatment stimulation (either verum or sham), the TBS-operator has to enter a six-digit numerical code—the patient key (for details see section “Randomization”)—into the stimulator. Depending on the orientation of the A/P coil, either the message "coil ready" or "turn coil" appears. The orientation of the coil with both sides looking alike determines whether TMS pulses target the brain (real) or the opposite direction (sham). Since in both conditions a “real TMS pulse” is triggered, the acoustic artefact is similar. The manufacturer will train the study staff in the operation of the simulator, and the principal investigator (CP) will train all study personnel operating TBS in determining treatment areas and coil position to ensure a consistent approach regarding stimulation procedure across all trial sites. All centers will also receive a training video.

#### Stimulation: electrical co-stimulation

The magnetic stimulation is accompanied by electrical co-stimulation to compensate for the typical somatosensory artefact and therefore ensure the greatest possible degree of blinding on the patient's side. For this purpose, flat electrodes (2 × 3 cm) will be attached to the patient's forehead: during left-sided iTBS, the center of the first electrode is fixed to FZ (EEG 10–20 system), the second electrode is fixed on the left forehead, i.e. rectangular aligned to the upper edge of the FZ-electrode. The distance between the edges of the two electrodes is 0.5 cm. During right-sided cTBS, the electrodes’ position is analogous with the second electrode on the right forehead, i.e. rectangular aligned to the upper edge of the FZ-electrode. The intensity of the co-stimulation is technically linked with the intensity of TBS. It is applied with 50% of the maximal output and, on this basis, automatically adjusted to the individual stimulation intensity in the active and the sham group.

#### Blinding

The use of the active/placebo coil in combination with the patient keys and electrical co-stimulation allows a double-blind design. As the patient key itself does not allow group assignment and the statistical center (IKEaB) is responsible for central randomization (for details see section “Randomization”), neither the patient, nor any study staff member is aware of group assignment. As an additional precaution, study staff members administering the ratings may not attend the stimulation sessions. Blinding for patients, all study staff members, and the biostatistician responsible for statistical analyses will be maintained until the data analysis is complete.

#### Concomitant treatment

Within the study, no specific antidepressant medication is mandatory. However, if present, the antidepressant therapy must be kept constant, which means that medication at or above the "minimum oral dose"(MOD) specified in the ATHF-SF [46] has to remain unchanged 4 weeks before and 6 weeks during study treatment. Changes below the MOD are considered as uncritical. Changes after the end of treatment are registered in the follow-up sessions. Critical changes during the treatment period lead to exclusion from the per protocol analysis. Antiepileptic drugs and/or benzodiazepines corresponding to > 1 mg lorazepam per day are not allowed during the trial. Accompanying psychotherapy is continued as specified in the individual treatment plan.

### Study endpoints

#### Primary outcome measure

We selected the difference in treatment response-rates (MADRS reduction of at least 50% of baseline value after the end of treatment period) between active combined iTBS/cTBS and the placebo condition as the primary endpoint. Thus, ambiguities regarding the clinical relevance of potentially small mean difference should be avoided and the results will derive a clear statement on the number to treat for a substantial treatment effect.

#### Secondary outcome measures

The considered secondary endpoints are (1) remission rate after treatment period which is defined as MADRS score ≤ 10 (2) comparison (active vs. sham) of raw score reduction of MADRS, HDRS-17, CGI, BDI-II, WHO-5, and WPAI during (a) treatment phase and (b) during follow-up (1 month and 3 months after end of treatment) compared to baseline for each time point; (3) number and severity of adverse events in both treatment arms and (4) deterioration rate after treatment which is defined as an increase of MADRS score of 25% compared to baseline.

### Sample size calculation

In an own pilot study (*n* = 32) we found 9/16 (56%) responders (MADRS ≤ 50% of baseline) in the verum group and 4/16 (25%) responders in the sham group [28]. In a second pilot study (*n* = 60) of another group [29] response rates (HDRS-17 ≤ 50% of baseline) were 66.7% vs. 13.3% after 2 weeks treatment and 4/15 (27%) vs. 1/15 (7%) after week 14. In the third pilot study (*n* = 56) [30] response rates (HDRS-17 ≤ 50% of baseline) were 55% (bilateral TBS) and 30% (sham TBS), 7 weeks after initiation of a 3 weeks treatment. In a recent large (total *n* = 385) clinical trial [18] comparing iTBS (*n* = 193) with 10 Hz rTMS (*n* = 192) the outcome criterion of HDRS-17 ≤ 50% of baseline was achieved by 49% of patients treated with iTBS. Based on these studies, we conservatively assume a response rate of 49% vs. 30% (combined bilateral TBS vs. sham TBS) using the criterion MADRS reduction of at least 50% compared to baseline. This leads to a sample size of 103 subjects per group (Chi-square test, alpha = 0.05 two-sided, beta = 0.2), totally 206 evaluable subjects. As we will recruit in seven centers we increase the sample size by *n* = 6 to compensate for degrees of freedom in a stratified analysis which leads to 212 patients. The analysis will be done on the full set of patients except for those refusing consent during the study or not receiving at least one treatment. We expect a low dropout rate and therefore, we increase the sample size by 24 subjects to adjust for 10% dropouts, which leads to a total n of 236 patients.

### Statistical analysis

Data of patients who receive at least one treatment will enter the primary intention-to-treat (ITT) analysis. The secondary per-protocol analysis (PP) will consider data of patients who participated in at least 80% (24/30) of the intended stimulation sessions.

Missing values will be imputed using multiple imputation approaches. The multiple imputation approach is used in the primary analysis. Complete case and last observation carried forward analysis will serve as sensitivity analysis. We expect to use monotone data imputation (missings caused by dropouts but not intermediate missing values in the time course) [47]. Thus natural predictors in the imputation model are preceding measurements of the outcome variable including baseline. Additionally, potential predictors as listed in the description of exploratory analyses will be included. Reproducibility will be ensured by the use of random seeds and variation due to the sampling process in the imputation will be reduced by a large number of imputation samples (*n* = 1000). The primary analysis will use a logistic regression model with MADRS ≤ 50% of baseline (yes/no) as dichotomous outcome, treatment arm as factor, baseline MADRS (continuous scale) and study center as covariate (factor). The goodness of fit for the logistic regression analysis will be inspected by use of the Hosmer–Lemeshow test. No interim analysis or planned subgroup analyses will be performed.

#### Secondary analyses

In a linear model, the outcome MADRS will be analysed as continuous variable. Covariates will be the same as in the primary analysis. Key secondary endpoints will be tested similarly with linear and logistic regression models. Additionally, mixed models will be applied to model the entire course of the primary and secondary outcomes during the study.

#### Exploratory analyses

Potential prognostic factors (disease duration, severity, degree of treatment resistance, age, sex) will be included in the multiple regression models with main effects and interactions (moderator effects) with treatment arm.

#### Descriptive analyses

They will include absolute and percentage frequencies for categorical variables, means, medians, standard deviations, quartiles and ranges for quantitative variables and medians, quartiles, and ranges for ordinal variables. Percentages will be estimated using an exact confidence interval for proportions based on the binomial distribution. The differences between subgroups will be tested using the Chi-square test (proportions), or the *t*-test for independent samples (normally distributed measures). Safety will be assessed by frequency tabulations, line listings, and exact 95% confidence intervals. All statistical analysis will be done using the software SPSS and R in the newest release.

### Documentation, monitoring and data management

With the exception of the data of the two satellite projects, all relevant data will be entered into a paper case report file (paperCRF) by responsible study team members. After monitoring, copies of the CRFs will be transferred to the central data management institution (see below). Each study center must store the original CRFs and other study-specific data for a minimum of 10 years.

The Center of Cinical Studies, University Tübingen (ZKS Tübingen) will monitor the implementation of the study with regard to correctness and Good Clinical Practice (GCP) conformity, which includes the review of the CRF entries in all trial sites. The main aims of the monitoring visits include the verification of the informed consent documents, existence of the patients, inclusion- and exclusion criteria, completeness and accuracy of entries on the CRFs (i.e. primary endpoint data). In each study center, the monitoring timeline includes an initiation visit, three visits per year and center during the recruitment period as well as a close-out visit after the complete documentation of the last patient/after clarification of queries by data management and/or monitoring.

The IKEaB is responsible for data management and will use Koordobas as a data management system, to which only authorized staff has password-protected access. After completion of monitoring of the CRF entries, the monitor will send a copy of the CRF to the data management. To ensure high data quality, IKEaB will implement double data entry by two different employees. Possible inconsistencies will be clarified by subsequent data reconciliation. At the same time, the data will be checked for completeness and plausibility. In case of queries, the data management sends a data query report to the respective center where responsible staff members will check original CRFs/source data for inconsistencies. After completion of data clearance, there will be a blind data review to determine the analysis population (intention-to-treat population, per-protocol population, and safety population).

### Safety aspects and Data Safety Monitoring Board

The exclusion criteria of this study include the currently valid contraindications for rTMS treatment [4]. In addition, a safety check (i.e. skin examination) is performed before each TBS session and immediately afterwards. Possible adverse events (AEs) or serious adverse events (SAEs) will be documented during the six-week treatment phase in accordance with the guidelines of GCP and will be followed up until complete recovery and/or the patient’s status is stable. AEs and SAEs must be reported to the monitoring facility and Data Safety Monitoring Board whose three members are independent and not affiliated with participating centers.

## Discussion

Lack of efficacy, insufficient tolerability or poor acceptance of common therapeutic approaches are major challenges in the treatment of MDD. Against this backdrop and based on comprehensive neurophysiological knowledge, rTMS has been established as an effective treatment option. Multiple clinical trials demonstrated and meta-analyses confirmed [3, 34, 48, 49] its efficacy in treating unipolar depression. However, particularly European treatment guidelines do not yet provide a clear recommendation for the application of rTMS [17]. Thus, clinicians are still hesitant in offering rTMS as a routine treatment option in MDD. Not the least, common rTMS treatment protocols are rather time-consuming and costly. With TBS, a substantially briefer and most likely at least similar effective [18, 28, 29] form of rTMS is available. However, although the recently documented non-inferiority of TBS compared to standard 10 Hz rTMS [18] is very valuable, it does not yet address the persisting doubts about the efficacy of rTMS in MDD treatment.

To meet that need, we designed this study (TBS-D) as a multicenter randomized placebo-controlled clinical trial to provide lacking evidence for a decisive recommendation regarding the use of rTMS in MDD treatment and to prove that 6 weeks of bilateral TBS—a particularly efficient and tolerable form of rTMS—is an effective add-on to standard MDD therapy. A positive result would primarily promote the adoption of rTMS for MDD treatment in general. Secondly, proof of the effectiveness of TBS would establish this faster and less burdening form of treatment. Thirdly, positive results would allow for more intensive treatment regimens like accelerated TBS [50] and thus path the way to a personalized treatment approach [20]. Regarding dropouts, we are confident to minimize them in particular because, the TMS operator sees the patient on a daily, the rater on a weekly basis. Hence, a strong bond between the study team and patient is established and the continuation of treatment is ensured. In addition, the well-known and robust placebo effect of TMS treatment should prevent dropouts in both treatment arms. However, for statistical analysis, we are aware of the risk of informative dropouts, depending on study arm or treatment success. A thorough analysis of patients who dropped out compared to patients with complete follow-up will be performed. The use of multiple imputations hopefully will adjust for this possible drawback.

In contrast to many of the large previous trials, the design of TBS-D aims to reflect clinical reality with TBS treatment as add-on to ongoing state-of-the-art therapy. To meet the clinical standards of MDD therapy, stimulation treatment should be integrated into a multimodal, individualized treatment concept. Therefore, proof of the efficacy of a stand-alone therapy with TBS would not have the required clinical relevance. Moreover, the inclusion of patients with mild to moderate treatment resistant MDD directs the focus on the majority of depressive patients. Based on these study characteristics we expect results with a high relevance for the further improvement of patient care and the development of treatment guidelines.

A specific challenge in NIBS-therapy, in general, is the multitude of stimulation parameters. For TBS this particularly affects stimulation intensity. Most of the pilot trials [28–30] were performed with intensities below the resting motor threshold (80% RMT) following the standard TBS procedure [22]. Nevertheless, the recent and so far largest non-inferiority trial [18] applied iTBS with 120% RMT, i.e. with a 50% higher stimulation intensity compared to standard TBS-procedure. This substantial increase was justified with reference to inadequate stimulation intensity in earlier rTMS trials. But for TBS it cannot be simply assumed that an increase in the intensity of standard 80% actually improves clinical efficacy by means of its risk/benefit ratio [51]. On the contrary, it has been shown that the TBS-effects on the motor-cortex do not linearly increase with intensity [52]. However, there are no indications that suggest a superior clinical efficacy of 120% compared to 80% TBS. In terms of clinical practicability, it is important to consider that an increase of stimulation intensity to 120% RMT also increases the stimulation-related discomfort and thus puts an additional burden on patients. Therefore, in the patients' interest and based on the pilot data, we decided to confirm the efficacy of 80% RMT in this trial.

Recent results indicate that both structural [53] and functional connectivity MRI parameters [54] may contain relevant information concerning TBS treatment responses in depression. Regarding the understanding of mechanisms and response predictors of TBS therapy, there is no doubt that individual network structure and activity [55, 56] as well as the genetic and epigenetic make-up [57] critically interacts with the clinical effect of TBS in depression. In the present study, we envisage to employ a multivariate pattern analysis approach for the prediction of TBS responses in depression [58] and to integrate the obtained parameters with the individual genetic and epigenetic make-up. Therefore, our study aims at providing comprehensive data for the identification of response predictors and in-depth analysis of high-dimensional, multimodal data sets including clinical, cognitive, genetic, epigenetic, and imaging data. In the future, this approach will allow for more precise planning and individualization of stimulation treatment.

In sum, positive results of TBS-D will give decisive support for the clinical use of bilateral TBS in the treatment of depression in addition to conventional standard therapy. This is all the more relevant since (bilateral) TBS with its substantially shorter treatment time compared to the rTMS standard treatment would qualify as the less burdening and more cost-effective approach. Finally, in synopsis with the clinical findings, the neuropsychological, imaging, and multi-omics data will extend our understanding of effect mechanisms and facilitate the individualization of antidepressant stimulation treatment.
